# Extra-Articular Manifestations in Reactive Arthritis due to COVID-19

**DOI:** 10.7759/cureus.18620

**Published:** 2021-10-09

**Authors:** Juan Camilo Santacruz, John Londoño, Ana María Santos, Angelo Arzuaga, Marta Juliana Mantilla

**Affiliations:** 1 Spondyloarthropathies Research Group, Universidad de la sabana, Chía, COL; 2 Spondyloarthropathies Research Group, Universidad de la Sabana, Chía, COL; 3 Rheumatology Department, Universidad Militar Nueva Granada, Bogotá, COL

**Keywords:** dactylitis, spondyloarthritis, extra-articular manifestations, covid 19, reactive arthritis

## Abstract

Reactive arthritis (ReA) is defined as arthritis that arises after infection, where pathogens cannot grow in the affected joints. Formerly, the clinical triad of postinfectious arthritis, urethritis, and conjunctivitis was called Reiter's syndrome; however, these clinical signs only represented a subset of patients with ReA. Due to the great diversity of its manifestations, its diagnosis is a challenge and can be overlooked in clinical practice. Additionally, it is associated with a variety of extra-articular manifestations that may be present either in the acute or chronic phase of the disease. Despite the cardinal clinical presentation characteristics of ReA, no case has been described in the literature that is diagnosed by the presence of classic extra-articular manifestations without objective joint involvement after COVID-19 infection. This report describes the case of a female patient in her third decade of life with an unusual presentation of ReA and focuses on her extra-articular manifestations.

## Introduction

The term "reactive arthritis" (ReA) was introduced in 1969 as an arthritis occurring shortly after or during an infection that did not occur in the joint itself but in other parts of the body without identification of the microorganism in the joint tissue [1]. The original definition did not specify the pathogens that were accepted as causes of ReA, and, in 1999, a panel of experts determined a specific list of gastrointestinal and urogenital pathogens that could be considered as potential causes, highlighting *Chlamydia trachomatis*, *Yersinia*, *Salmonella*, *Shigella*, and *Campylobacter* [2]. Formerly, the clinical triad of post-infectious arthritis, urethritis, and conjunctivitis was called Reiter's syndrome; however, these clinical signs represented only a subset of patients with ReA [3]. Also, bacterial respiratory infections caused by *Chlamydia psittaci*, *Chlamydia pneumoniae*, *Staphylococcus aureus*, and *Streptococcus pneumoniae*, or by viral infections (such as COVID-19) have been associated with certain cases of ReA [4]. The disease typically debuts as an asymmetric monoarthritis or oligoarthritis, affecting mainly the lower extremities (ankles and knees), and usually presents one to three weeks after the triggering infection [5]. Due to the great diversity of its manifestations, its diagnosis is challenging and may be overlooked in clinical practice [6]. Although there is no consensus regarding the diagnostic criteria for ReA, in most cases it is based on the association of clinical and microbiological factors. Additionally, it is associated with a variety of extra-articular manifestations that may be present either in the acute or chronic phase of the disease [7]. It should be noted that the frequency of the latter has not been analyzed in detail, except in a European cohort of 186 patients where the frequencies of ocular and cutaneous involvement were 20% and 15%, respectively [8]. Despite the cardinal clinical features of ReA presentation, no case has been described in the literature that is diagnosed by the presence of its classic extra-articular manifestations without objective joint involvement subsequent to COVID-19 infection. The following describes the case of a female patient in the third decade of life with an unusual presentation of ReA centered on its extra-articular manifestations.

## Case presentation

**Figure 1:**
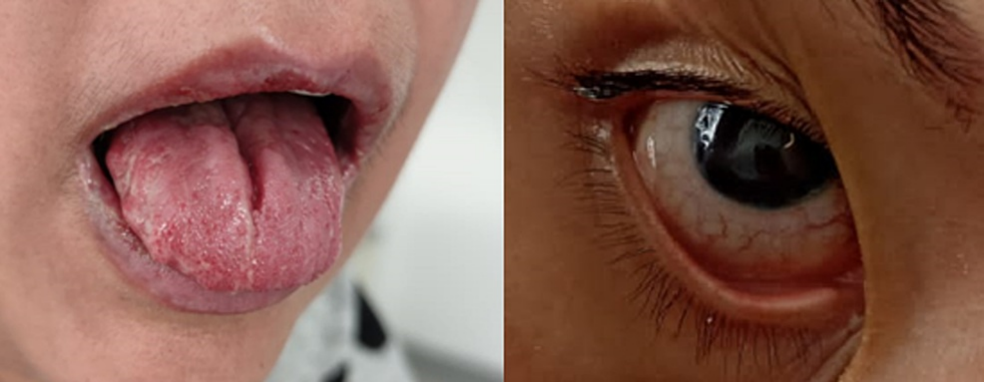
Conjunctivitis and oral lesions in reactive arthritis The figure on the left shows gray plaques on the tongue with some ulcers on the lower lip and labial commissure. The figure on the right shows the presence of conjunctival injection, a characteristic finding of conjunctivitis.

**Figure 2:**
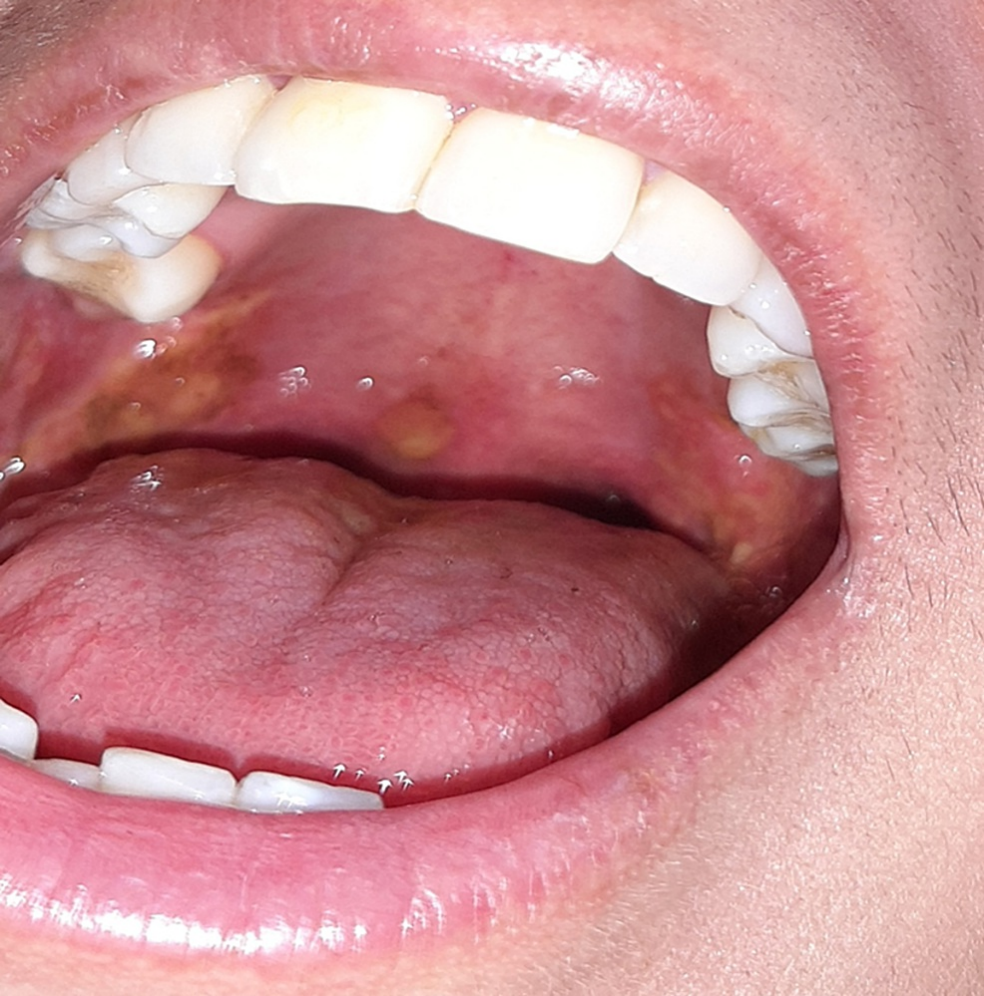
Palatal erosion in reactive arthritis A sharply demarcated erosion of the hard palate is shown.

A 30-year-old female patient from Maracaibo (Venezuela) with a history of severe respiratory infection by COVID-19 for four months presented with clinical symptoms of six days consisting of odynophagia associated with anosmia, dysgeusia, bilateral conjunctival injection, fever of 38.5 degrees Celsius, and dyspnea. She had a report of a positive antigen test and biochemical markers of poor prognosis as evident by lymphopenia, and increased lactic dehydrogenase, C-reactive protein, transaminases, and D-dimer. She received treatment with dexamethasone according to the RECOVERY study protocol for mild oxygenation disorder for four days without progression to pulmonary phase IIB and was discharged at that time. She presented again two months later due to decreased visual acuity and bilateral ocular pain of left predominance of one month associated with the appearance of painful oral aphthous ulcers involving the lips, palate, and tongue (Figures 1, 2).

**Figure 3:**
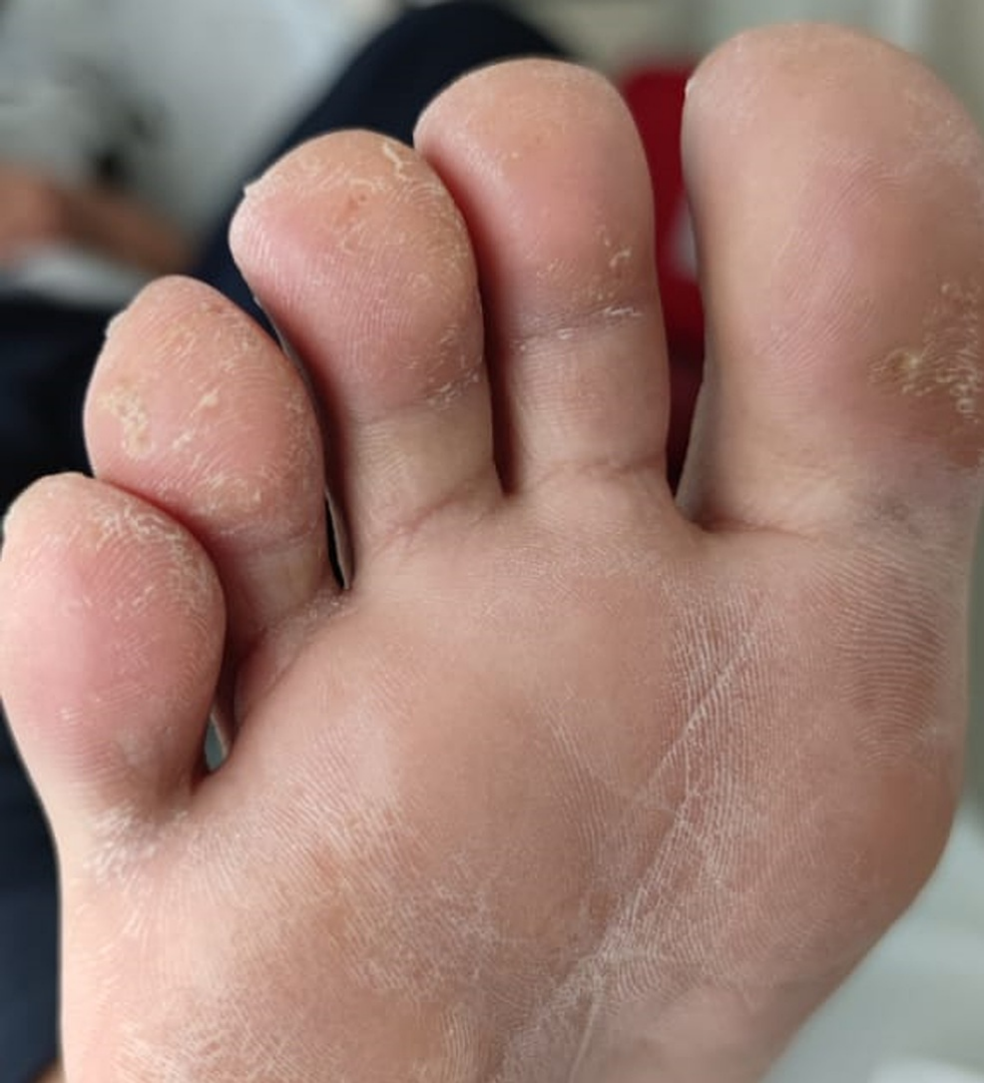
Blennorrhagic keratoderma in reactive arthritis Hyperkeratotic skin lesions are seen on the soles of the feet corresponding to a blennorrhagic keratoderma

**Figure 4:**
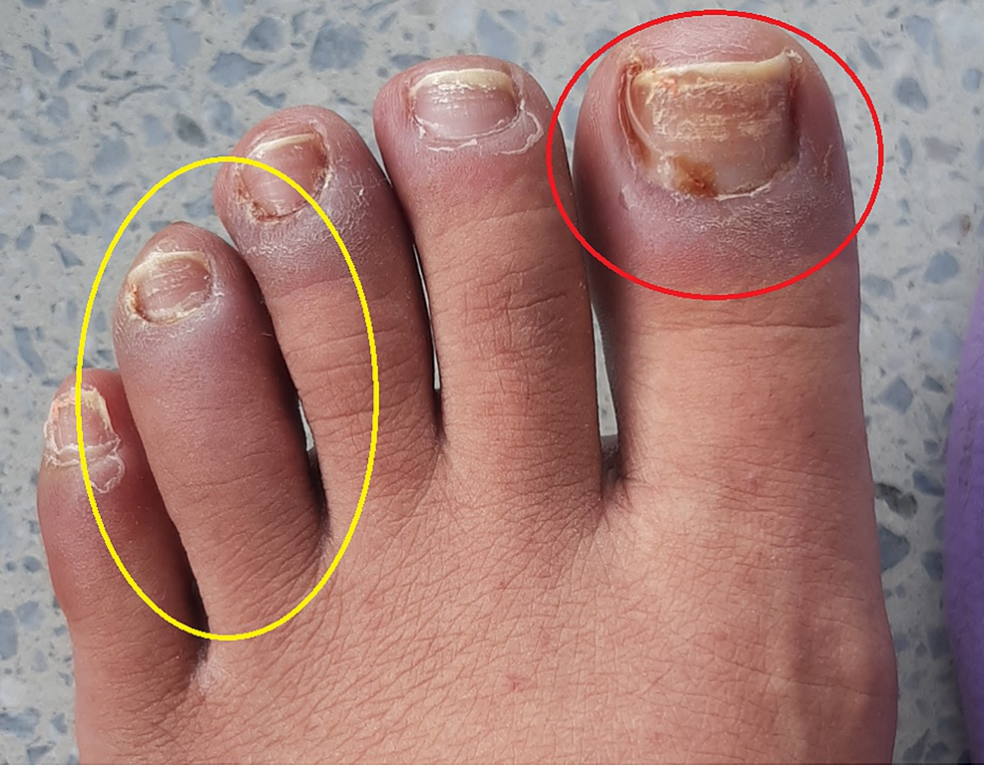
Nail changes and psoriasiform lesions in reactive arthritis Dactylitis of the fourth toe (yellow oval) with subungual hyperkeratosis and onycholysis (red oval) is seen on the left foot.

Additionally, skin lesions with psoriasiform characteristics were documented on the soles of the feet with changes suggestive of subungual hyperkeratosis in the nails and dactylitis of the fourth toe of the left foot (Figures 3, 4).

 

**Figure 5:**
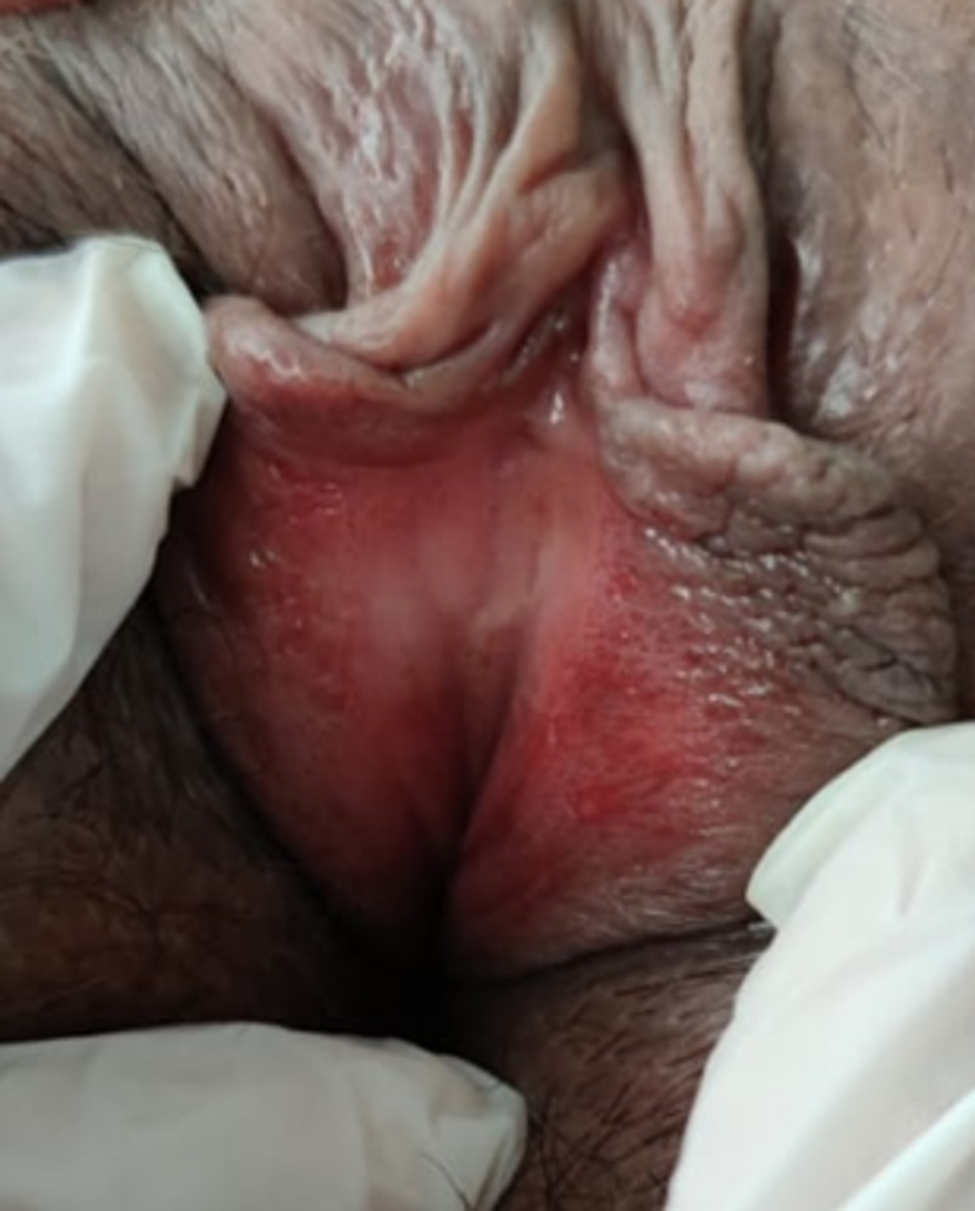
Circinate vulvitis in reactive arthritis An image corresponding to circinate vulvitis with erythematous lesions on the vaginal mucosa with linear ulcers at the bottom of the folds of the labia majora can be seen.

During the course of both hospitalizations, the presence of monoarthritis or oligoarthritis, inflammatory lumbar pain, or enthesitis was not established. She mentioned that she received outpatient treatment with nystatin, clotrimazole, itraconazole, fluconazole, and prednisolone, reporting partial improvement only with the latter at a dose of 15mg per day. On review by systems, she reported the appearance of painful ulcers on the labia majora associated with occasional vaginal discharge, and therefore the assessment was complemented with a smear of vaginal discharge with no signs suggestive of infection and on gynecological assessment with no clinical changes suggestive of cervicitis (Figure 5).

The patient was evaluated by rheumatology who supported the absence of joint involvement; however, semiologically she met the classic characteristics of extra-articular manifestations of ReA with involvement of the ocular and cutaneous system without achieving a significant improvement until that moment. The description of the most representative paraclinical studies upon admission and during hospitalization is shown in Table 1.

**Table 1:** Laboratory parameters at admission and prior to discharge HIV, human immunodeficiency virus; VDLR, Venereal Disease Research Laboratory; CRP, C-reactive protein; LVEF, left ventricular ejection fraction; Ig, immunoglobulin; HLA, human leukocyte antigen

Paraclinical studies	Upon admission	Prior to discharge
Hemoglobin (g/dL)	16.3	14.3
Mean corpuscular volume (fL)	95.5	90
Total leukocyte count (/µl)	6,960	3,470
Total lymphocyte count (/µl)	1,410	1,840
Total platelet count (/µL)	369,000	151,000
Creatinine (mg/dL)	0.38	0.45
Ureic nitrogen (mg/dL)	8.8	3.3
Sodium (meq/L)	132	138
Potassium (meq/L)	3.83	3.57
Magnesium (meq/L)	1.64	-
HIV	Non-reactive	-
VDRL test	Non-reactive	-
CRP (mg/dL)	-	40.6
Transthoracic echocardiogram	Normal (LVEF: 55-60%)	-
*Mycoplasma pneumoniae* IgG	1.0	-
*Mycoplasma pneumoniae* IgM	1.5	-
*Chlamydia trachomatis* IgG	2.7	-
*Chlamydia trachomatis* IgM	5	-
HLA classes I and II	A2, A23, B15, B27, DR1, and DR14	-
Vaginal discharge smear	Negative for infection	-
Uroanalysis	Negative for infection	-

## Discussion

As previously mentioned, ReA rarely presents without articular manifestations. In fact, oligoarticular involvement of the disease is the most frequent form of presentation in 70% of women and 73% of men, followed by monoarticular and polyarticular involvement [9]. Low back pain and "sausage finger" dactylitis are also clinical features of the initial presentation [10]. Among the extra-articular manifestations, genitourinary symptoms such as urethritis, cervicitis, salpingo-oophoritis, cystitis, and prostatitis stand out [11]. Regarding ocular symptoms, conjunctivitis is observed with a frequency close to 51% in cases of acute ReA. In the chronic phase, uveitis occurs more frequently and is the most common ocular symptom [12]. The most frequently observed dermatological manifestations are aphthous ulcers (in up to 60%), circinate balanitis (10-40%), blennorrhagic keratoderma (in more than 20%), and rarely erythema nodosum [13-14]. Onycholysis and nail pits occur in approximately 10% of cases [14]. At present, no case of ReA has been described that debuts only by its extra-articular manifestations, and several of them occur at the same time after infection by COVID-19. Several cases of ReA have been described in the world related to COVID-19, presenting mostly with arthritis and dactylitis, but no extra-articular manifestations, except for one case that presented with transient synovitis in the right elbow and psoriasis plaques in the same location two weeks after infection [15]. It has been proposed that some patients with COVID-19 present a state of transient immunosuppression with the consequent predisposition to certain immune-mediated diseases, inducing an inflammatory response with marked elevation of IL-17 (interleukin-17), which is fundamental in the pathophysiology of ReA, psoriasis, and psoriatic arthritis [16]. In fact, high plasma levels of IL-17 have been described in patients with Middle East respiratory syndrome, and patients with COVID-19 have increased numbers of circulating Th17 cells [17]. Other studies have confirmed higher levels of IL-17 along with other pro-inflammatory cytokines such as tumor necrosis factor (TNF), interferon-γ, and IL-2 [18]. It should be noted that treatments targeting extra-articular manifestations have not been systematically evaluated in randomized studies or have been the subject of observational studies. Topical glucocorticoids have been proposed in cases of mild cutaneous involvement or methotrexate and anti-TNF in cases of severe blennorrhagic keratoderma associated with pustular lesions [19]. For the treatment of oral aphthous ulcers, only symptomatic treatment, or in some cases topical glucocorticoids, have been indicated since in most cases they are self-limited. Our patient achieved prompt remission of all her extra-articular manifestations with intermediate doses of glucocorticoids (prednisolone 15mg per day).

## Conclusions

It can be concluded that COVID-19 infection has had very heterogeneous cases of ReA presentation, particularly the one we presented, debuting with extra-articular manifestations without evident arthritis. It is important to know in detail these manifestations and their frequency of presentation since none of them is included within the classification criteria, and the diagnosis was reached by its description and semiological support. It is likely that in relation to COVID-19 infection, these manifestations occur more frequently in the acute phase of the disease and require a more intensive immunomodulatory treatment. Additionally, this is the only case reported with HLA (human leukocyte antigen) B27 and B15 positivity, which could imply a risk in the development of true chronic spondyloarthritis, present features of other spondyloarthritis such as psoriatic arthritis, or represent a risk factor for the genesis of extra-articular manifestations of ReA. A better understanding of the immune alterations that accompany SARS-CoV-2 (severe acute respiratory syndrome coronavirus 2) infection may represent a useful opportunity to further investigate the immunopathogenic mechanisms capable of promoting the development of extra-articular manifestations in ReA. According to current knowledge of immunopathogenesis, blocking the IL-17 pathway could be a promising therapeutic alternative in these cases.
